# Supplementary material for: Definition of RNA Polymerase II CoTC Terminator Elements in the Human Genome
Source: Cell Rep. 2013 Apr 25;3(4):1080–92. doi: 10.1016/j.celrep.2013.03.012 (PMC3644702; doi:10.1016/j.celrep.2013.03.012)
Supplement: Table S1. RNA Linker and DNA Primer List, Related to Experimental Procedures [file mmc1.pdf]

Table S1. RNA Linker and DNA Primer List

Primer	Sequence 5'-3'	information
**CLIP-seq**		
RL5	5′-OH AGG GAG GAC GAU GCG G 3′-OH	5' RNA linker (GA IIX), made by Thermo
RL3	5′-P GUG UCA GUC ACU UCC AGC GG 3′-puromycin	3' RNA linker (GA IIX), made by Thermo
DP5	AGGGAGGACGATGCGG	5' PCR primer (GA IIX)
DP3	CCGCTGGAAGTGACTGACAC	3' PCR primer (GA IIX)
RL5_solexa1	5'-N6 ACACGACGCUCUUCCGAUCUrNrNrNAA -3'OH	5' RNA linker with barcode NNNAA (Hiseq2000), made by Thermo
RL5_solexa2	5'-N6 ACACGACGCUCUUCCGAUCUrNrNrNCC -3'OH	5' RNA linker barcode NNNCC (Hiseq2000), made by Thermo
RL3_solexa	5'-p AGAUCGGAAGAGCGGUUCAG -3'Puromycin	3' RNA linker(Hiseq2000), made by Thermo
P5solexa	AATGATACGGCGACCACCGAGATCTACACTCTTTCCCTACACGACGCTCTTCCGATCT	5' PCR primer(Hiseq2000)
P3solexa	CAAGCAGAAGACGGCATACGAGATCGGTCTCGGCATTCCTGCTGAACCGCTCTTCCGATCT	3' PCR primer(Hiseq2000)
**PCR**		
β5'pA	GGGATATTATGAAGGGCCTTGAGC	
β3'pA	GAACTAGCTCTTCATTTCTTTATG	
7SK_FW	GACATCTGTCACCCCATTGA	
7SK_RV	GCCTCATTTGGATGTGTCTG	
CCNB1_F1	AGCTAGTATCTTCAAACTTTC	
CCNB1_R1	CCATAATTGATGTCCTTCAGG	
AKIRIN1_F1	TGTCTTTGACTTAAATCTAAC	
AKIRIN1_R1	AATGGTTATAGCAGCTCTCAC	
PTCH2_F1	TTACTAAAGATTTCCGTGGAG	
PTCH2_R1	AACCTCCGTCTTCTAGAGTCA	
THOC2_F1	AGCATTGTCTTATGAAATGCT	
THOC2_R1	AGTTCAGCAATACAAAGCTCC	
WDR13_F1	ATGTCAGCTTCAACTGCGACG	
WDR13_R1	TTCCACCGTGTTCCTGGAGCT	
GAPDH_F1	CGCACCTTGTCATGTACCATC	
GAPDH_R1	TTCCTGTAGCACTCAAGACGT	
PKM2_F1	TTGCTATAGAGCCTACCTGTA	
PKM2_R1	ACCAGGAGGTGGGCAGGATGG	
ENO1_F1	ACTTTCCACCAAGTGTCTAGA	
ENO1_R1	CAATGGATCCTCATCCTTTTC	
BF	CCTTGGGAAAATACACTATATC	
BR	CTTGAATCCTTTTCTGAGGGATG	
BETAtermF	CATCCCTCAGAAAAGGATTCAAG	
BETAtermR	GAAACCATACCTCTACTGTCT	
BR2	GATGCGTAAGGAGAAAATACC	
CCNB1_F2	GTCCCATAAGATTATGATGCC	
CCNB1_F3	GTCTTGAACACCCGACCTCAG	
CCNB1_F4	GCTTGTCCAACTTGAAATATC	
CCNB1_F5	CTCACTATGTTGTCCAAGCTG	
CCNB1_R2	TACTCACACAAATCTAGATGG	
CCNB1_R3	TATGTCATCTGGTTCTATCAA	
CCNB1_R4	GTGTTCAGTAGTACTCCCATA	
CCNB1_R5	AACTGATGTCAACTGGATAAG	
CCNB1_R2.1	GAGTGAGACTCTTGTCTCAAA	

Primer	Sequence 5'-3'	information
WDR13_F2	ACAAGCATTGATTGAATGTCT	
WDR13_F3	CATGTTGCTCAGGCTGGTCTT	
WDR13_F4	ATGATCTGACTTGGTCATAGA	
WDR13_F5	TGTCAAAGTTACCTGAAGCAG	
WDR13_R2	CAGTGCCTAGAACAGTTCCTG	
WDR13_R3	ACTCACTTCATTCAGGTCTTT	
WDR13_R4	CCTGCAGCCTAACATAGTGAA	
WDR13_R5	GTTCAGCAGCTCAGCAATGCT	
THOC2_R2	TAAGCACTCAACAGTCACTGA	
THOC2_R3	CACAAATATTTACTATCTGGC	
THOC2_R4	TGACTAGAATGGAAGTTCCAT	
THOC2_R5	TCAGAGTTAGTTATAAAGGTC	
PTCH2_R2	CAAACCGCTAACAGCTACTGT	
PTCH2_R3	GCCTGTAATCCCAGATGCTGA	
PTCH2_R4	AGTTAGGGGCTATTGCAATTA	
PTCH2_R5	CTGGGAGTTTGATACCAGTTC	
AKIRIN1_R2	CTGCCTTAGGAGCTCTGATCT	
AKIRIN1_R3	AGCAAGTTTAGTAATCTGGCA	
AKIRIN1_R4	TCTCCAGGTCTGACGAAGACT	
AKIRIN1_R5	CTACTCAAGTCTGGAGTTAGT	
**Cloning**		
BETA43	CTTGAATCCTTTTCTGAGGGATG	
BETA10.3	CAGACAGTAGAGGTATGGTTTCCAGGG	
CCNB1termF	CCATCTAGATTTGTGTGAGTA	For PCTE with CCNB1_R4
AKIRIN1termF	AGATCAGAGCTCCTAAGGCAG	For PCTE with AKIRIN1_R4
PTCH2termF	ACAGTAGCTGTTAGCGGTTTG	For PCTE with PTCH2_R4
THOC2termF	GCCTTGCAGGCCATGTAGTTC	For PCTE with THOC2_R5
WDR13termF	GGAGCTGCTAAGACTTAGGGA	For PCTE with WDR13_R4
