# Supplementary material for: Definition of RNA Polymerase II CoTC Terminator Elements in the Human Genome
Source: Cell Rep. 2013 Apr 25;3(4):1080–92. doi: 10.1016/j.celrep.2013.03.012 (PMC3644702; doi:10.1016/j.celrep.2013.03.012)
Supplement: Document S1. Article plus Supplemental Information [file mmc2.pdf]

Cell PRESS
Open ACCESS

Cell Reports
Article

Definition of RNA Polymerase II CoTC Terminator Elements in the Human Genome

Takayuki Nojima,[1] Martin Dienstbier,[2] Shona Murphy,[1] Nicholas J. Proudfoot,[1,*] and Michael J. Dye[1,*]
[1]Sir William Dunn School of Pathology, University of Oxford, South Parks Road, OX1 3RE Oxford, UK
[2]MRC Functional Genomics Unit, Department of Physiology, Anatomy and Genetics, University of Oxford, South Parks Road, OX1 3QX Oxford, UK
*Correspondence: nicholas.proudfoot@path.ox.ac.uk (N.J.P.), michael.dye@path.ox.ac.uk (M.J.D.)
http://dx.doi.org/10.1016/j.celrep.2013.03.012

SUMMARY

Mammalian RNA polymerase II (Pol II) transcription termination is an essential step in protein-coding gene expression that is mediated by pre-mRNA processing activities and DNA-encoded terminator elements. Although much is known about the role of pre-mRNA processing in termination, our understanding of the characteristics and generality of terminator elements is limited. Whereas promoter databases list up to 40,000 known and potential Pol II promoter sequences, fewer than ten Pol II terminator sequences have been described. Using our knowledge of the human β-globin terminator mechanism, we have developed a selection strategy for mapping mammalian Pol II terminator elements. We report the identification of 78 cotranscriptional cleavage (CoTC)-type terminator elements at endogenous gene loci. The results of this analysis pave the way for the full understanding of Pol II termination pathways and their roles in gene expression.

INTRODUCTION

The transcription cycle consists of three stages. Initiation, where RNA polymerase engages with the DNA template, followed by elongation where it translocates along the DNA template synthesizing the RNA copy of the gene and finally termination, where polymerase disengages from the DNA template. In mammals, promoter and terminator DNA sequences are well described for genes transcribed by RNA polymerases I and III (Richard and Manley, 2009). For genes transcribed by RNA polymerase II (Pol II), including all protein coding genes, there is an extensive literature describing promoter sequences. The Mammalian Promoter Database lists 24,967 known and an additional 17,926 potential human Pol II promoters (Gupta et al., 2011); however, fewer than ten verified mammalian Pol II terminator sequences have been described (Proudfoot, 1989; Richard and Manley, 2009).

The major reason for this discrepancy is that Pol II termination is coupled to the complex steps involved in pre-mRNA processing and is entirely dependent upon the presence of a functional poly(A) signal (Whitelaw and Proudfoot, 1986; Connelly and Manley, 1988). Pre-mRNA cleavage at the poly(A) site, mediated by the 3′ end processing complex (Shi et al., 2009), generates two RNA products; a 5′ cleavage product that is stabilized by polyadenylation as it is processed into mRNA and a 3′ cleavage product that is subject to rapid degradation by the 5′-3′ exonuclease Xrn2. Such Xrn2-mediated transcript degradation has been shown to have a role in Pol II termination (West et al., 2004). The above could lead one to conclude that the poly(A) site sequence, typically characterized by the hexanucleotide sequence AATAAA followed by a GU/U rich region (Proudfoot, 2011), is the sole Pol II terminator signal. That this is indeed the case, in lower eukaryotes, is supported by a number of studies, mainly in yeast, which provide a detailed understanding of the roles of a host of RNA processing factors in the Pol II termination process (Richard and Manley, 2009; Kuehner et al., 2011). For mammalian genes, however, the poly(A) signal is not the only sequence that is required for Pol II termination. Studies in our laboratory and others have shown the existence of dedicated DNA-encoded Pol II terminator elements located downstream of poly(A) signals (Proudfoot, 1989; Tantravahi et al., 1993; Dye and Proudfoot, 2001; Plant et al., 2005; Gromak et al., 2006; West et al., 2006). From these studies, it appears that there are two broad categories of terminator sequence; G-rich sequences that enhance poly(A) site cleavage and subsequent Pol II termination by pausing Pol II near to the poly(A) signal (Proudfoot et al., 2002; Gromak et al., 2006) and AT-rich terminator sequences, located 1–2 kb downstream of the poly(A) site, which mediate rapid cotranscriptional cleavage of nascent transcripts, prior to poly(A) site cleavage (Dye and Proudfoot, 2001; Plant et al., 2005; West et al., 2006). RNA degradation initiating at cotranscriptional cleavage (CoTC) sites leads to release of Pol II and associated unprocessed pre-mRNA, from the DNA template (West et al., 2008). This distinguishing feature of CoTC-mediated termination is supported by electron microscopic studies in *Drosophila* that show that release of pre-mRNA from transcription sites prior to 3′ end processing is a common occurrence (Osheim et al., 2002).

One of the major reasons for the paucity of mammalian Pol II terminator sequences in the literature is that examination of termination mechanisms is hindered by the technical difficulty of mapping nascent transcripts in nuclear run on experiments

(Proudfoot, 1989). Here we have used a CLIP-seq strategy (Licatalosi et al., 2008) to distinguish pre-mRNAs that are released from the DNA template before cleavage at the poly(A) site, in order to identify genes that utilize the CoTC termination pathway. Thorough testing of potential terminator sequences from a number of candidate gene loci shows that we have isolated authentic Pol II terminators and indicates that CoTC-mediated termination is a feature of a significant proportion of mammalian genes.

RESULTS

CLIP-seq-Based CoTC Terminator Mapping Strategy

Detailed transcriptional analysis of the human β-globin gene has shown that CoTC of β-globin 3′ flanking region transcripts leads to release of Pol II and associated pre-mRNA, from the chromatin template prior to cleavage/polyadenylation at the poly(A) site. Interestingly, 3′ end processing of these released pre-mRNAs (referred to herein as unprocessed pre-mRNAs) occurs posttranscriptionally, in the nucleoplasm (Figure 1A) (West et al., 2008). Following on from these observations, we have developed a strategy, using in vivo UV crosslinking immunoprecipitation (IP) with antibody to the CstF-64 pre-mRNA processing factor (MacDonald et al., 1994), to select such unprocessed nucleoplasmic pre-mRNAs in order to identify genes that use the CoTC termination pathway.

In an initial pilot experiment, HeLa cells transiently transfected with a β-globin minigene construct βTERM (that has an HIV-LTR promoter) and a plasmid encoding the viral transactivator Tat (pTat), were subjected to UV crosslinking followed by nuclear fractionation into chromatin (Ch) and nucleoplasm (N) fractions, as described previously (West et al., 2008). IP was then conducted on the nucleoplasm fraction using CstF-64 antibody to precipitate unprocessed β-globin pre-mRNA, which was detected by RT-PCR using β-globin and control 7SK snRNA primers (Figure 1B). In lane 1, the detection of PCR products representing unprocessed β-globin pre-mRNA and mature 7SK transcripts, confirms the presence of both RNA species in the input nucleoplasm fraction. In control lane 2, no PCR products are detected confirming that 7SK and β-globin pre-mRNA do not interact with IgG. In lane 3, RT-PCR of nucleoplasmic RNA precipitated with the CstF-64 antibody shows the presence of a PCR product for β-globin but not 7SK, confirming a specific interaction of CstF-64 with nucleoplasmic β-globin pre-mRNA.

We next performed CstF-64 IP, combined with high throughput sequencing (CLIP-seq) (Licatalosi et al., 2008), to identify endogenous CstF-64 interacting nucleoplasmic pre-mRNAs. HeLa cells transiently transfected with βTERM and pTat were UV crosslinked prior to nuclear fractionation. The nucleoplasm fraction was then treated with high (40 U/ml; lanes 1–4) or low (4 U/ml; lanes 5–8) RNaseI before IP with CstF-64 and IgG antibodies. Immunoprecipitated RNA was 5′ end-labeled with [γ-^{32}P] ATP and protein RNA complexes were separated by PAGE and transferred to a nitrocellulose membrane (Figure 1C). IP of UV-treated cells with CstF-64 antibody (lanes 4 and 8) results in two prominent radiolabeled bands. (The absence of bands in control lanes 1–3 and 5–7, confirms the specificity of the CstF-64 IP experiment; see legend for full details). The lower 70 kDa band is the expected size for CstF-64 and the upper ~200 kDa band is possibly a CstF-64 dimer. The bands detected in lane 8 (4 U/ml) are relatively weaker than those detected in lane 4 (40 U/ml) and appear within a radioactive smear extending from 40–300 kDa, which reflects partial RNA digestion due to limiting RNaseI. RNA was eluted from the membrane at a position 20–30 kDa above the 70 kDa CstF-64 band to obtain CstF-64/RNA complexes containing 50–80 nucleotide (nt) RNAs. Eluted RNAs were ligated to adaptors for reverse transcription and PCR amplification. This experiment was repeated twice more on untransfected cells. PCR products from each experiment were analyzed by high throughput sequencing (HITS). A total of 1,285 CLIP regions were identified, from the three repeats, which were supported by at least two independent read alignments in the pooled samples. These CLIP regions were significantly enriched in genic (RefSeq+RNA genes) but not intergenic regions and within genes, they showed significant enrichment in extended 3′ UTR regions, but not in exons or introns (graphs, Figure 1D). Seventy-eight genes (Table 1) containing CstF-64 CLIP regions within 3′ UTRs (i.e., in proximity to putative poly(A) sites) were selected as CoTC candidates (see Experimental Procedures for details). Importantly, this list contains the β-globin gene, as three unique CLIP reads were mapped in the proximity of its annotated poly(A) site, in the transfected sample (Figure 1E).

CLIP-seq Positive Candidate Pre-mRNAs Are Released from the DNA Template

According to our hypothesis, the detection of a particular unprocessed pre-mRNA in the nucleoplasm is a marker of CoTC-type termination occurring at the corresponding gene locus. Therefore, to further test the CLIP-seq data, we analyzed the nuclear distribution of unprocessed pre-mRNA, from a subset of CLIP-seq positive genes, by quantitative radioactive RT-PCR (qRT-PCR) of chromatin and nucleoplasm fractions (Figure 2A). Analysis of pre-mRNA in the chromatin (Ch) fraction (lane 1) shows that unprocessed, presumably nascent, pre-mRNAs are detected at each of the candidate gene loci. Likewise, for the nucleoplasm (N) fraction (lane 2), unprocessed pre-mRNA from each of the candidate gene loci is detected. Quantitative analysis of the PCR products, in lanes 1 and 2, confirms that significant amounts of unprocessed pre-mRNA (19%–55%) are released to the nucleoplasm fraction from the corresponding gene loci (Figure 2A, graph). Variation in the amount of nucleoplasmic pre-mRNA, from different genes, may indicate variation in the efficiency of pre-mRNA release, gene-specific rates of 3′ end processing or differential RNA stability. Importantly, these data confirm our detection of unprocessed nucleoplasmic pre-mRNAs in our CLIP-seq analysis and support the hypothesis that a CoTC-type termination mechanism operates at these gene loci.

To control for the possibility that the presence of unprocessed nucleoplasmic pre-mRNA is a general feature of Pol II transcribed genes, we employed the same qRT-PCR strategy to examine the nuclear distribution of *GAPDH*, *PKM2*, and *ENO1* pre-mRNAs, as these highly expressed transcripts (Kapranov et al., 2007) were not detected in our CLIP-seq analysis. As shown in Figure 2B, analysis of pre-mRNAs in the chromatin

Cell PRESS Open ACCESS

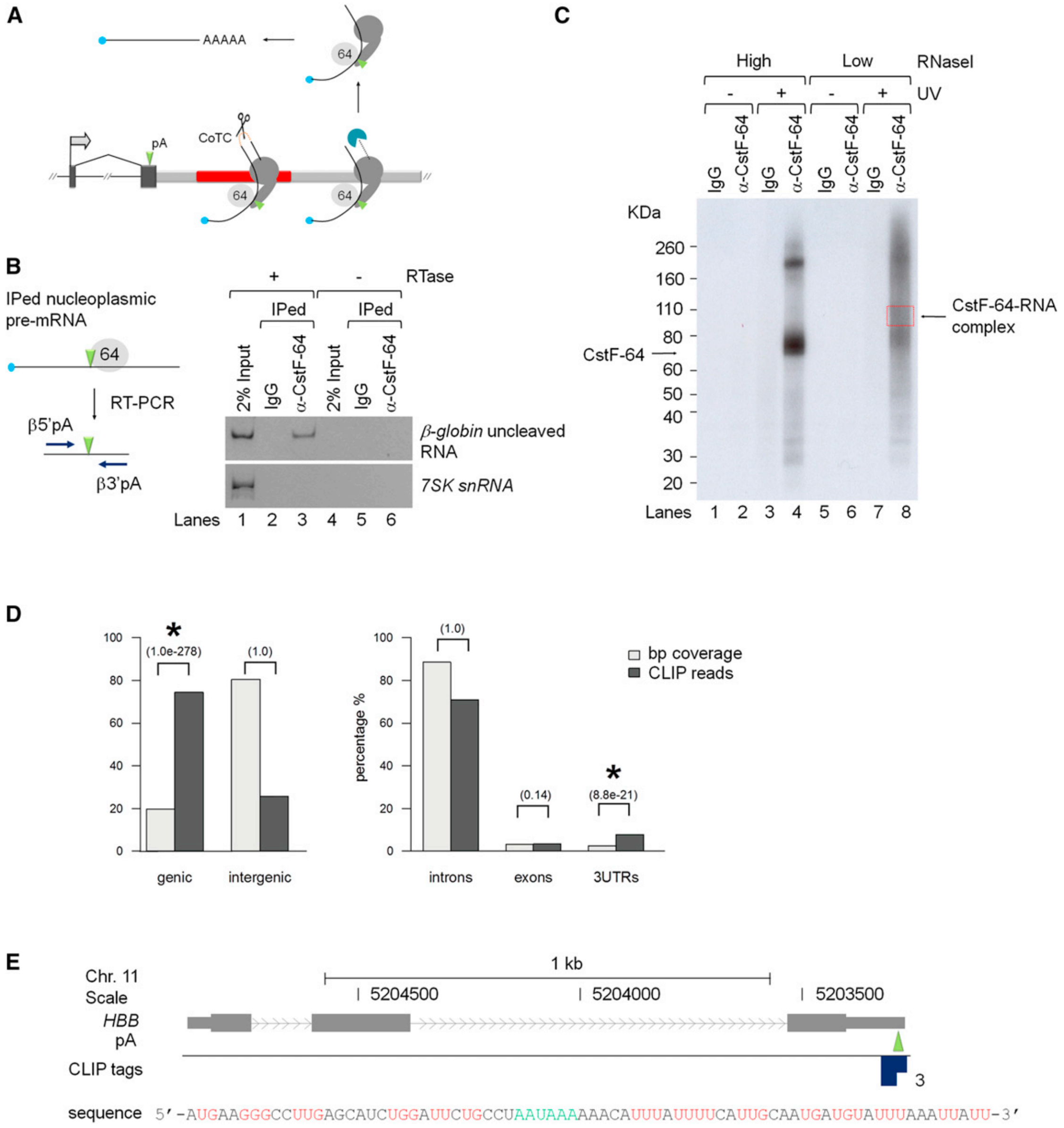

Figure 1. Isolation of Nucleoplasmic CstF-64-Associated Pre-mRNA

(A) Diagram of the CoTC termination mechanism. The CoTC terminator (red bar) is located downstream of the poly(A) site (pA, green arrowhead) of the human β-globin gene. β-globin pre-mRNA (curved black line with blue cap) is generated by Pol II (gray icons). 3′ flanking region transcripts (red dashed line) are cleaved by CoTC activity (scissors). Xrn2 (blue icon) degrades the 3′ transcript (black dashed line) leading to Pol II and pre-mRNA release from the DNA template. Pre-mRNA is processed by poly(A) factors including CstF-64 (gray sphere) in the nucleoplasm to mRNA (line with AAAA indicating poly(A) tail).

(B) RT-PCR analysis of immunoprecipitated nucleoplasmic RNA. RNA in control input (lanes 1 and 4) and immunoprecipitated with rabbit IgG (lanes 2 and 5) or CstF-64 antibody (lanes 3 and 6) was subjected to RT-PCR analysis using β-globin-specific primers (blue arrows in diagram beside the data panel) and primers for 7SK snRNA. The lack of bands in lanes 4–6 (−RTase) confirms the absence of contaminating DNA in all samples.

(C) Purification of CstF-64-RNA covalent complexes by SDS-PAGE following treatment with high (40 U/ml, lanes 1–4) or low (4 U/ml, lanes 5–8) RNaseI. No protein-RNA complexes were detected in the absence of UV irradiation (lanes 1, 2, 5, and 6) or following control immunoprecipitation with rabbit IgG (lanes 3 and 7), demonstrating the specificity of the CstF-64 antibody/CstF-64-RNA complex interaction. The red dashed box, in lane 8, indicates the area of the gel from which CstF-64-RNA complexes were eluted for subsequent HITS analysis.

(D) Bar graphs showing the distribution of CLIP reads in annotated genic and intergenic regions (left panel) and in intron, exon, and 3′ UTRs of annotated genes (right panel). P values (brackets) for the significance of relative enrichment, with respect to what would be expected by chance, in the case of a random distribution, were calculated using a one-sided binomial test. *Indicates significant enrichment (p value < 0.001).

(E) Diagram of the human β-globin gene with CLIP tags (dark blue squares) mapped to the sequence shown below the diagram. The annotated poly(A) site is indicated in green. Red letters highlight G/U stretches representing potential CstF-64 binding sites.

(Ch) fraction (lane 1) detected unprocessed, presumably nascent, pre-mRNAs at each of the candidate gene loci. In lane 2, analysis of the nucleoplasm fraction detected only faint PCR products representing unprocessed nucleoplasmic pre-mRNAs from these gene loci. Quantitative analysis of the radiolabeled RT-PCR products derived from chromatin and nucleoplasm fractions (graph, Figure 2B) shows that unprocessed nucleoplasmic pre-mRNAs represent merely 4%–7% of total pre-mRNA from these gene loci. These data provide further validation of the CLIP-seq experiment and indicate that, on the basis of our selection criteria (detection of unprocessed nucleoplasmic pre-mRNA), it is likely that the *GAPDH*, *PKM2*, and *ENO1* genes do not employ a CoTC termination mechanism. We predict that the termination mechanism employed at these gene loci is similar to the previously described pause-type, where poly(A) site cleavage precedes Pol II release from the DNA template (Gromak et al., 2006; West et al., 2008) (diagram, Figure 2B).

Mapping of Terminator Elements at CLIP-seq Positive Gene Loci

We next developed a strategy to map terminator elements at the CLIP-seq positive gene loci. Transcript cleavage occurring downstream of and prior to cleavage at the poly(A) site is an intrinsic part of the CoTC terminator mechanism (Dye and Proudfoot, 2001; West et al., 2008) (Figure 1A). Therefore we reasoned that it would be possible to infer the location of potential CoTC terminator sequences, in the 3′ flanking regions of CLIP-seq positive genes, by mapping sites of transcript cleavage using an RT-PCR approach. Total nuclear RNA was reverse transcribed using random primers and the resulting cDNA was PCR amplified using gene-specific primers complementary to 3′ flanking region transcripts. For *CCNB1*, PCR amplification was carried out using a single forward primer (F), located upstream of the *CCNB1* poly(A) site, in combination with reverse primers (R1–R5) located at increasing distance downstream of the poly(A) site in the *CCNB1* 3′ flanking region (Figure 3A, diagram). PCR amplification using the F/R1-R3 primer pairs resulted in the detection of 180 bp, 580 bp, and 1.1 kb bands (lanes 6–8), which correspond precisely to bands derived from control amplification of genomic DNA using the same primer pairs (lanes 1–3). PCR amplification using F/R4 and F/R5 primer pairs does not result in detectable levels of product (lanes 9 and 10), even though corresponding PCR products are derived from control amplification of genomic DNA (lanes 4 and 5). Thus RT-PCR analysis shows that continuous *CCNB1* pre-mRNA is detected up to ~1.1 kb downstream of the poly(A) site (lane 8, F/R3 primer pair), with no continuous RNA detected beyond this point (lanes 9 and 10). From these data, we estimated that a potential CoTC terminator element (PCTE) was located between 470–1,780 bp downstream of the *CCNB1* poly(A) site (the region bordered by primers R2 and R4). This observation is similar to that reported for the β-globin gene, where significant CoTC activity occurs at a position ~1.0 kb downstream of the β-globin poly(A) site (Dye and Proudfoot, 2001). We next adopted the same terminator mapping strategy for four other CLIP-seq positive genes (*AKIRIN1*, *PTCH2*, *THOC2*, and *WDR13*). Transcript cleavage (3′ flanking region) was mapped to positions 0.8–1.2 kb downstream of the respective poly(A) sites and was used to estimate the location of PCTEs (Figure 3B). Confirmation of these results comes from control experiments, measuring RT-PCR efficiency on full-length in vitro transcribed *CCNB1*, *PTCH2*, and *WDR13* 3′ flanking region transcripts (Figure S1).

Testing Candidate Terminator Elements

The five newly identified PCTEs were placed in the termination reporter plasmid (βΔTERM) and tested for terminator activity by RNase protection assay (RPA) (Plant et al., 2005). Nuclear RNA isolated from HeLa cells transiently transfected with each of the candidate reporter constructs, positive (βTERM), or negative (βΔTERM) terminator control constructs and pTat, was hybridized to an antisense radiolabeled riboprobe spanning the reporter gene HIV-LTR (Figure 4A). Following RNase digestion, protected products were analyzed by PAGE (Figure 4B). In lane 1, no protection products were detected, confirming the absence of β-globin mRNA in untransfected HeLa cells. In lane 2 (βTERM), the prominent 85 nt band, (labeled mRNA) at the base of the gel, results from hybridization of the riboprobe to the 5′ end of the β-globin mRNA. The weaker 242 nt band (labeled RT) results from hybridization of the riboprobe to read-through transcripts derived from Pol II transcription proceeding around the plasmid into the HIV-LTR. In lane 3 (βΔTERM), a weaker mRNA band was detected together with a prominent read-through band that reflects increased Pol II read-through transcription in the absence of the β-globin terminator element. In lane 4 (βCCNB1), the reduced intensity of the read-through band shows that the *CCNB1* PCTE effectively blocks Pol II read-through transcription. Furthermore, the corresponding increase in the mRNA band shows that β-globin mRNA recovers to wild-type level in the presence of the *CCNB1* PCTE. The low level of the read-through band in all candidate PCTE samples (lanes 5–8 and graph below the data panel) indicates that each PCTE has terminator activity.

Although RPA is useful for screening terminator activity, it is an indirect method and read-through transcript levels could be affected by differential RNA stability. Therefore, we next employed nuclear run on (NRO) analysis to measure termination by the *CCNB1* and *PTCH2* PCTEs. NRO analysis was conducted on nuclei isolated from HeLa cells transfected with βCCNB1, βPTCH2, and three control constructs (βTERM, βΔTERM, and β4-7) along with pTat. Resulting radiolabeled nascent transcripts were hybridized to the nylon filter shown in Figure 4C. For the positive control construct (βTERM) prominent hybridization signals are detected in the gene body and post poly(A) site region (probes P, B3, and B4, respectively) and background level signals are detected over probes A and U3, which are located downstream of the terminator, showing that efficient termination occurs before Pol II reaches region A of the plasmid template (Dye and Proudfoot, 2001). For the termination negative control (βΔTERM), in the absence of the terminator sequence, hybridization signals are detected over all probes P-U3. The presence of prominent signals over probes A and U3 shows that in the absence of the terminator Pol II transcribes the entire plasmid. The transcription profiles resulting from NRO analysis of βCCNB1 and βPTCH2 are essentially identical to βTERM, with prominent radioactive signals detected over probes P, B3 and B4 and only background

Cell PRESS
Open ACCESS

Table 1. List of CLIP-Seq-Positive CoTC Candidate Genes

Gene Symbol	Transcript_ID	Gene Name
AGFG1	NM_004504	ArfGAP with FG repeats 1
AKIRIN1	NM_001136275	akirin 1
BCAP29	NM_001008405	B cell receptor-associated protein 29
BLCAP	NM_006698	bladder cancer-associated protein
BRD9	NM_001009877	bromodomain containing 9
C5orf28	NM_022483	chromosome 5 open reading frame 28
C5orf35	NM_153706	chromosome 5 open reading frame 35
CANX	NM_001746	Calnexin
CCDC72	NM_015933	coiled-coil domain containing 72
CCNB1	NM_031966	cyclin B1
CD55	NM_001114752	CD55 molecule, decay accelerating factor for complement (Cromer blood group)
CELF1	NM_001172640	CUGBP Elav-like family member 1
CHST15	NM_015892	carbohydrate (N-acetylgalactosamine 4-sulfate 6-O) sulfotransferase 15
CYBRD1	NM_024843	cytochrome b reductase 1
CYFIP1	NM_014608	cytoplasmic FMR1 interacting protein 1
DDX3X	NM_001356	DEAD (Asp-Glu-Ala-Asp) box polypeptide 3, X-linked
DDX58	NM_014314	DEAD (Asp-Glu-Ala-Asp) box polypeptide 58
DMD	NM_004019	Dystrophin
DNAJC6	NM_014787	DnaJ (Hsp40) homolog, subfamily C, member 6
FAM200B	NM_001145191	family with sequence similarity 200, member B
FAR1	NM_032228	fatty acyl CoA reductase 1
GNAI3	NM_006496	guanine nucleotide binding protein (G protein), α inhibiting activity polypeptide 3
GNB2L1	NM_006098	guanine nucleotide binding protein (G protein), β polypeptide 2-like 1
HADHA	NM_000182	hydroxyacyl-Coenzyme A dehydrogenase/3-ketoacyl-Coenzyme A thiolase/enoyl-Coenzyme A hydratase (trifunctional protein), α subunit
HBB	NM_000518	hemoglobin, β
HES7	NM_001165967	hairy and enhancer of split 7 (*Drosophila*)
HN1L	NM_144570	hematological and neurological expressed 1-like
HNRNPD	NM_031369	heterogeneous nuclear ribonucleoprotein D (AU-rich element RNA binding protein 1, 37 kDa)
HNRNPM	NM_031203	heterogeneous nuclear ribonucleoprotein M
IRX3	NM_024336	iroquois homeobox 3
KCMF1	NM_020122	potassium channel modulatory factor 1
KLF6	NM_001160125	Kruppel-like factor 6
MAFK	NM_002360	v-maf musculoaponeurotic fibrosarcoma oncogene homolog K (avian)
MARK3	NM_001128920	MAP/microtubule affinity-regulating kinase 3
MIB1	NM_020774	mindbomb homolog 1 (*Drosophila*)
MRPL30	NM_145212	mitochondrial ribosomal protein L30
MRPS14	NM_022100	mitochondrial ribosomal protein S14
MYC	NM_002467	v-myc myelocytomatosis viral oncogene homolog (avian)
NCS1	NM_001128826	frequenin homolog (*Drosophila*)
NQO2	NM_000904	NAD(P)H dehydrogenase, quinone 2
NUCKS1	NM_022731	nuclear casein kinase and cyclin-dependent kinase substrate 1
NUFIP2	NM_020772	nuclear fragile X mental retardation protein interacting protein 2
OMA1	NM_145243	OMA1 homolog, zinc metallopeptidase (*Saccharomyces cerevisiae*)
PRPF4B	NM_003913	similar to hCG1820375; PRP4 pre-mRNA processing factor 4 homolog B (yeast)
PSIP1	NM_021144	PC4 and SFRS1 interacting protein 1
PTCH2	NM_001166292	patched homolog 2 (*Drosophila*)
RAB13	NM_002870	RAB13, member RAS oncogene family; similar to hCG24991

(*Continued on next page*)

Table 1. Continued

Gene Symbol	Transcript_ID	Gene Name
RAPH1	NM_213589	Ras association (RalGDS/AF-6) and Pleckstrin homology domains 1
RBM27	NM_018989	RNA binding motif protein 27
RNASEH1	NM_002936	ribonuclease H1
RNF166	NM_178841	ring finger protein 166
RPL13A	NM_012423	ribosomal protein L13a pseudogene
RPL37A	NM_000998	ribosomal protein L37a
RRP15	NM_016052	ribosomal RNA processing 15 homolog (*S. cerevisiae*)
SAC3D1	NM_013299	SAC3 domain containing 1
SAP18	NM_005870	Sin3A-associated protein, 18 kDa
SCAF8	NM_014892	RNA binding motif protein 16
SCLT1	NM_144643	sodium channel and clathrin linker 1
SIK1	NM_173354	salt-inducible kinase 1
TEAD1	NM_021961	TEA domain family member 1 (SV40 transcriptional enhancer factor)
TFAP2A	NM_001042425	transcription factor AP-2 α (activating enhancer binding protein 2 α)
THOC2	NM_001081550	THO complex 2
TNRC6A	NM_014494	trinucleotide repeat containing 6A
TROAP	NM_001100620	trophinin-associated protein (tastin)
TUBA1B	NM_006082	hypothetical gene supported by AF081484; NM_006082; tubulin,α 1b
TXLNG	NM_001168683	γ-taxilin
TXNRD1	NM_001093771	thioredoxin reductase 1; hypothetical LOC100130902
WDR13	NM_001166426	WD repeat domain 13
WDR36	NM_139281	WD repeat domain 36
WNK1	NM_014823	WNK lysine deficient protein kinase 1
YWHAZ	NM_145690	tyrosine 3-monooxygenase/tryptophan 5-monooxygenase activation protein, ζ polypeptide
ZAK	NM_133646	sterile α motif and leucine zipper containing kinase AZK
ZBTB9	NM_152735	zinc finger and BTB domain containing 9
ZDHHC7	NM_001145548	zinc finger, DHHC-type containing 7
ZNF215	NM_013250	zinc finger protein 215
ZNF616	NM_178523	zinc finger protein 616
ZNF714	NM_182515	zinc finger protein 714
ZNF740	NM_001004304	zinc finger protein 740

List of nonredundant RefSeq protein-coding genes containing CstF-64 CLIP regions (supported by at least two read-alignments) within poly(A) site regions (extended 200 nt downstream). These genes represent candidates for the CoTC termination mechanism. ‘‘Ribonucleoprotein complex’’ and ‘‘RNA binding’’ GO-TERMs were significantly enriched (Benjamini < 0.05) among these genes (DAVID Bioinformatics Resources). HBB derives from CLIP1 where cells were transfected with the β-globin expression plasmid βTERM.

level signal over probes A and U3. NRO analysis of control construct β4-7, which includes an 850 bp spacer sequence in place of the terminator, confirms that the effect of *CCNB1* and *PTCH2* PCTEs on the NRO profile is due to the presence of authentic Pol II terminator signals and not to arbitrary spacing effects.

The *CCNB1* Terminator Mediates CoTC Activity

Having confirmed that a subset of PCTEs mediate efficient Pol II termination, we next conducted further analysis of two PCTEs to measure CoTC activity. Detailed analysis of the human β-globin CoTC terminator shows that cotranscriptional cleavage of terminator transcripts precedes Pol II disengagement from the DNA template (Figure 1A) (Dye and Proudfoot, 2001; West et al., 2008). To determine if a similar order of events occurs at the *CCNB1* terminator, we began by measuring the distribution of transcribing Pol II in the endogenous *CCNB1* terminator region, by qRT-PCR analysis of nascent transcripts. Total nuclear RNA was reverse transcribed using random primers and the resulting cDNA was PCR amplified, using primer pairs to detect transcripts of the *CCNB1* poly(A) site and terminator regions (see upper diagram, Figure 5A). The resulting PCR products were quantified by PhosphoImage analysis before plotting on the graph (gray bars, Figure 5A). PCR amplification, using primer pair F1/R1, results in a prominent signal reflecting the high abundance of transcripts in the poly(A) site region. PCR amplification of cDNA representing *CCNB1* terminator transcripts, using primer pair F2/R2, indicates relatively high transcript abundance at the 5′ end of the terminator. However, transcript abundance is significantly decreased by the middle of the terminator, primer

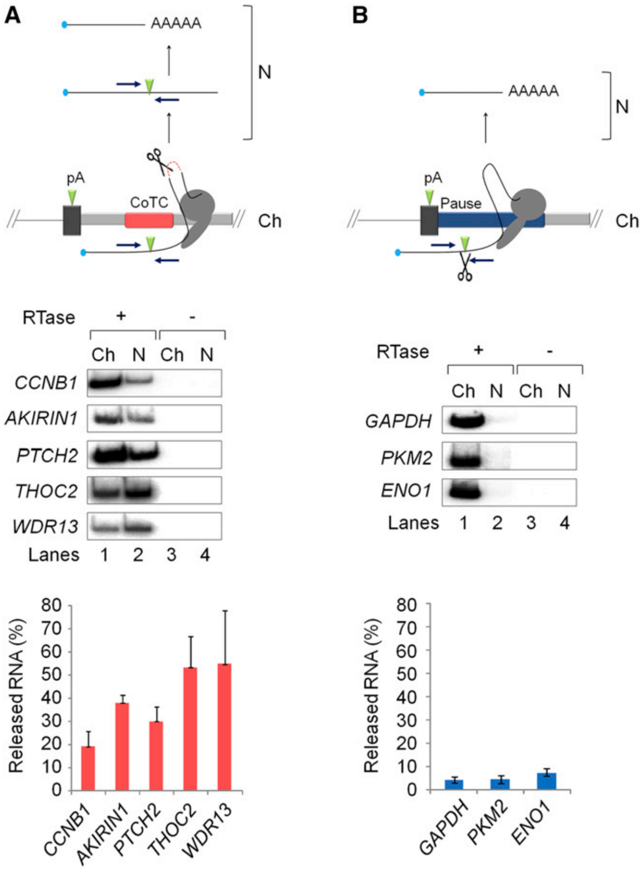

Figure 2. Nuclear Distribution of Pre-mRNA from CstF-64 CLIP Positive and Negative Gene Loci

CLIP positive (A) and negative (B) gene names are shown to the left of the corresponding data panels. RT-PCR analysis was performed on pre-mRNA from chromatin (Ch, lanes 1 and 3) and nucleoplasm (N, lanes 2 and 4) fractions. cDNA synthesis was primed using random primers, in reactions with (+RTase, lanes 1 and 2) or without (−RTase, lanes 3 and 4) the addition of reverse transcriptase to control for the presence of contaminating DNA. PCR amplification (23 cycles) of the resulting cDNA was conducted using gene-specific primers (indicated by blue arrows in the diagrams above the data panels) spanning annotated poly(A) sites (UCSC Genome Browser; http://genome.ucsc.edu/). (The number of PCR cycles was determined to be within the linear range [data not shown] and therefore accurately reflects RNA abundance). Radiolabeled RT-PCR products from chromatin and nucleoplasm fractions (lanes 1 and 2) were quantitated by PhosphoImage analysis and the proportion of released nucleoplasmic pre-mRNA (% total) calculated and displayed in the graphs below the data panels. The lack of PCR products in lanes 3 and 4 (−RTase) confirms the absence of contaminating DNA in all samples. The diagrams above the data panels illustrating CoTC-type (A) and pause-type (B) termination mechanisms are labeled as Figure 1A, except for the pause element (blue bar) and scissors (indicating cleavage by the 3′ end processing complex) in (B). Error bars represent the results of three experimental repeats.

pair F3/R3, falling to ~10% at the 3′ end of the terminator element (primer pair F4/R4). Examination of the post-terminator region, using primer pair F5/R5, indicates a further decrease in active Pol II level. These data show that the level of transcriptionally engaged Pol II decreases as it proceeds through the terminator region, indicative of Pol II termination. We next examined the continuity of *CCNB1* terminator transcripts by qPCR of random primed cDNA, using primer pairs composed of a single forward primer (F1), located immediately upstream of the *CCNB1* poly(A) site, in combination with five different reverse primers (R1–R5; see lower diagram, Figure 5A). The resulting radioactive PCR products were quantified by PhosphoImage analysis before plotting on the graph (black bars, Figure 5A). This analysis shows that whereas high levels of continuous transcripts are detected with reverse primers positioned before the terminator element, very few or none (7%–0%) are detected with primers positioned within or downstream of the terminator element. These data show that nascent transcripts of the 5′ end of the *CCNB1* terminator are cotranscriptionally cleaved and, when combined with results from the measurement of transcript distribution above, indicate that transcript cleavage precedes Pol II termination occurring throughout the *CCNB1* terminator. The profile of transcript discontinuity followed by Pol II termination is similar to that described for the human β-globin gene terminator (Dye and Proudfoot, 2001) and is indicative of the presence of the CoTC termination mechanism at the *CCNB1* gene locus. To test if other candidate genes utilize the CoTC termination pathway we conducted analogous qRT-PCR terminator transcript analysis on the endogenous *WDR13* gene and observed terminator transcript discontinuity occurring before Pol II termination, again indicating the presence of the CoTC termination mechanism (Figure S2).

Terminator and CoTC Activities Localize to the 5′ End of the *CCNB1* Terminator Element

To analyze the *CCNB1* terminator in more detail, it was divided into three subfragments (labeled A, B, and C) that were placed in the reporter plasmid βΔTERM, forming βCCNB1A, βCCNB1B, and βCCNB1C (diagram, Figure 5B). The termination capacity of each subfragment was compared to that of the full-length *CCNB1* terminator (in βCCNB1) by RPA. Nuclear RNA isolated from HeLa cells transiently transfected with βCCNB1/A/B and C, positive (βTERM) and negative (βΔTERM) control constructs and pTat, were hybridized to an antisense radiolabeled riboprobe spanning the HIV-LTR (Figure 5B). The positive (βTERM, lane 2) and negative (βΔTERM, lane 3) controls show that βTERM promotes efficient Pol II termination, as indicated by the reduced read-through signal in lane 2. In lane 4 (βCCNB1), restoration of read-through and mRNA protection products, to the levels seen with the termination positive control βTERM (lane 2), confirms that the full *CCNB1* terminator mediates efficient Pol II termination. In lane 5 (βCCNB1A) a similar pattern of low read-through and high mRNA signal is observed, indicating that region A of the *CCNB1* terminator mediates efficient Pol II termination. In lanes 6 (βCCNB1B) and 7 (βCCNB1C), the significantly lower level of the mRNA band and higher level of the read-through band indicates that regions B and C of the *CCNB1* terminator have reduced terminator activity. This observation is confirmed by quantitative phosphoImage analysis of the radiolabeled protection products, shown in the graph below the data panel.

We next tested the *CCNB1* terminator subfragments for CoTC activity by measuring transcript abundance using qRT-PCR. Nuclear RNA isolated from HeLa cells transiently transfected with βCCNB1/A, B, or C and pTat, was reverse transcribed with PCR primers labeled BR and terR, which are

Cell PRESS
Open ACCESS

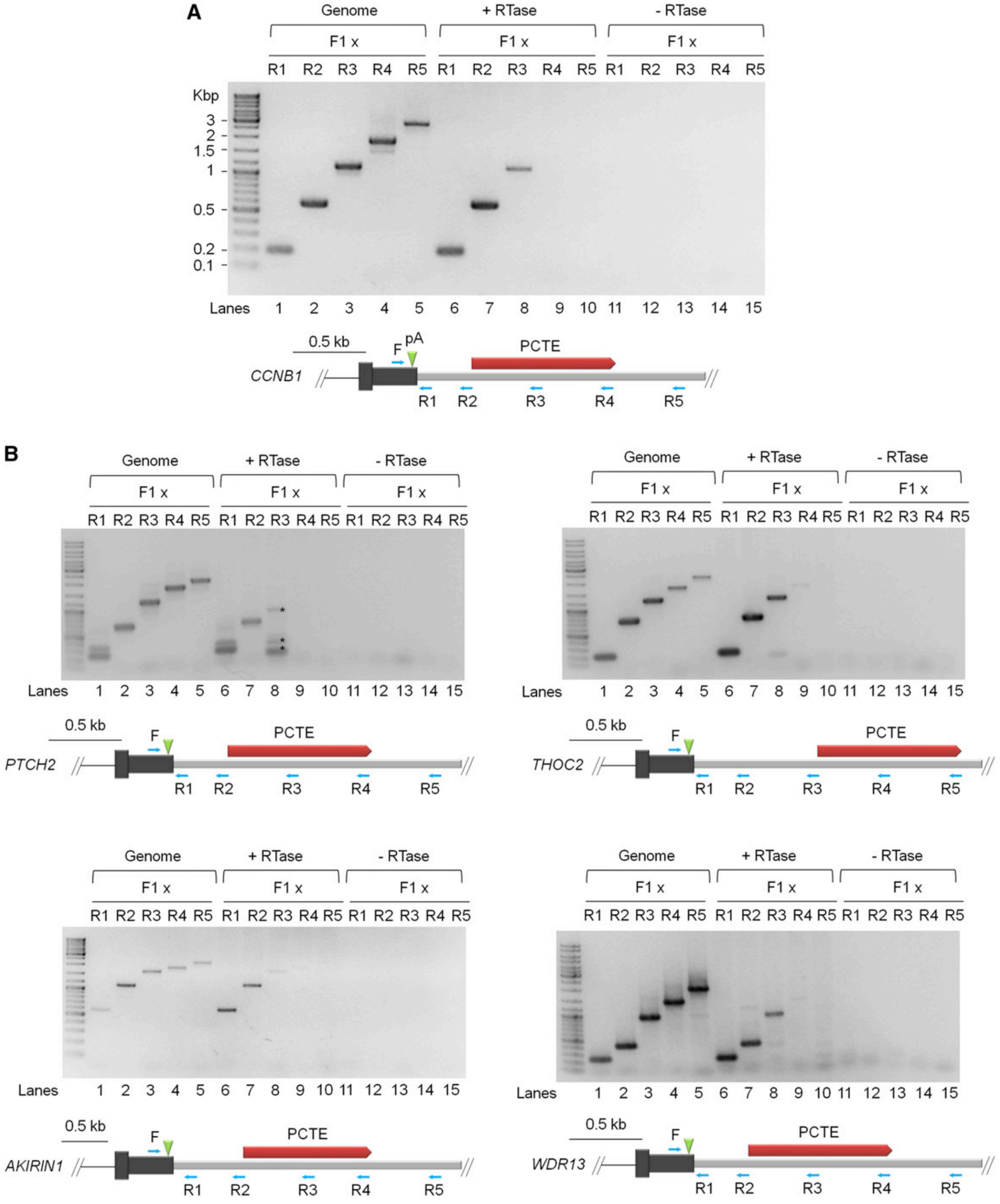

Figure 3. Mapping CoTC Terminator Elements at Endogenous Gene Loci

(A) RT-PCR analysis of *CCNB1* 3′ flanking region transcripts using primer pairs indicated (blue arrows) in the diagram below the data panel. Lanes 1–5, control PCR amplification of genomic DNA. Lanes 6–10 (+RTase) PCR amplification of reverse transcribed *CCNB1* 3′ flanking region transcripts. Lanes 11–15, control PCR amplification of −RTase samples. The diagram below the data panel shows the *CCNB1* 3′ UTR (gray box), poly(A) site (green arrowhead), and putative CoTC terminator element (PCTE, red bar).

(B) RT-PCR analysis of *PTCH2*, *THOC2*, *AKIRIN1*, and *WDR13* 3′ flanking region transcripts. Sample lanes and diagram notation are as in (A). *In PTCH2 lane 8, indicates nonspecific RT-PCR products of unknown origin. Control PCR amplification (−RTase, lanes 11–15) confirms the absence of contaminating DNA. See also Figure S1.

complementary to plasmid sequence either side of the inserted *CCNB1* terminator subfragments (diagram, Figure 5C). We then conducted PCR on the resultant cDNA using primer pairs BF/BR and terF/terR to analyze transcripts from upstream of and across the terminator subfragments (Figure 5C). The absence of PCR products in lane 1 confirms that there is no

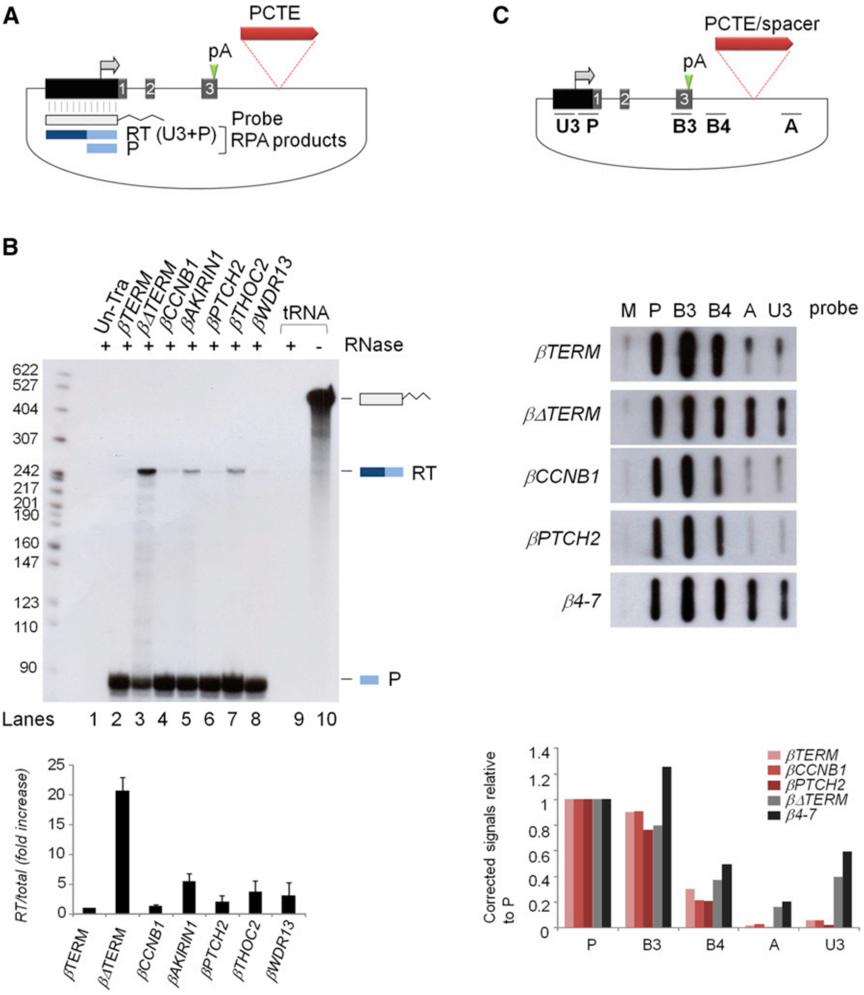

Figure 4. Measuring Terminator Efficiency

(A) Diagram of the βΔTERM construct. The transcription start site (gray arrow), poly(A) site (green arrowhead, labeled pA), insertion site of PCTEs, and β-globin terminator (red bar) are indicated. The HIV-LTR antisense riboprobe (tailed rectangle), expected readthrough (RT), and mRNA (P) protection products (blue bars) are also shown.

(B) RNase protection termination assay (RPA). Lane 1, untransfected cells, lanes 2–8 transfected cells. Control RNase digestion of the riboprobe is shown in lane 9 (tRNA$^+$) beside undigested riboprobe (tRNA$^-$, lane 10). For each sample RT and P protection products were quantified by PhosphoImage analysis and the relative abundance of the RT product (RT/Total) was calculated and displayed in the graph below the data panel. Error bars represent the results of three experimental repeats.

(C) Nuclear run on (NRO) analysis of *CCNB1* and *PTCH2* terminator elements. In the diagram, above the data panel, positions of single strand (ss) DNA probes, with respect to the plasmid template, are indicated by characters in bold. Slot blots in the data panel show the distribution of nascent transcripts from the plasmid templates indicated to the left of the panel. The bar graph, below the data panel, shows quantitation of NRO hybridization signals after removal of background hybridization signal, detected by control probe M (M13 DNA), and correction for [α-^{32}P]UTP content of the hybridized nascent transcripts.

background signal in untransfected HeLa cells. In lane 2 (βCCNB1A) amplification with the BF/BR primer pair yields a prominent PCR product, representing transcripts from upstream of terminator subfragment A. However, PCR amplification of the region A transcript, with the terF/terR primer pair, results in a very low abundance PCR product indicating that few continuous RNA transcripts extend across this region. In contrast, βCCNB1/B and C generated prominent PCR products with both primer pairs, indicating that abundant continuous RNA transcripts extend across these subfragments of the CCNB1 terminator. The very low level of RT-PCR product representing transcripts of subfragment A indicates that they are subject to CoTC. The correspondence of the robust terminator activity of subfragment A, shown by RPA (Figure 5B), and the observed discontinuity of its transcript, shown both here (lane 2, Figure 5C) and in qRT-PCR of terminator transcripts from the endogenous gene locus (Figure 5A), contrasts with the weak terminator activity and apparent stability of region B and C transcripts. These data provide further compelling evidence that Pol II termination on the *CCNB1* gene is mediated by the CoTC termination mechanism and confirm that, using the CLIP-seq strategy, we have successfully identified authentic Pol II terminator elements.

Bioinformatic Analysis of CoTC Terminator Sequences

In order to search for conserved DNA or RNA sequences involved in the CoTC termination mechanism we next conducted a detailed bioinformatic analysis of the 3′ flanking regions (0–2 kb downstream of the CLIP-seq sites) of all 78 CoTC candidate genes. From this analysis we found that candidate gene 3′ flanking regions are slightly more AT- and T-rich than equally sized regions downstream of the annotated pA sites of other protein coding genes (Figure S3). This finding is in agreement with our analysis of the β-globin terminator that has shown the importance of AT rich sequences in CoTC-mediated termination of β-globin gene transcription (Dye and Proudfoot, 2001; White et al., 2013). Next, in an effort to identify possible *trans*-acting factors in the CoTC termination process, we screened the candidate gene 3′ flanking regions for the presence of DNA binding motifs of known transcription factors represented in the professional version of the TRANSFAC database, which includes 1,665 binding motif matrices. We found that none of these motifs were significantly enriched or depleted in the tested set when compared to the corresponding region of other protein coding genes. Finally, we conducted a search for potential novel sequence motifs by using MEME software (Bailey and Elkan, 1994). Although, as expected, some weak motifs were identified in a subset of candidate gene 3′ flanking regions using this approach (see Figure S4), their relevance to

Cell
PRESS
Open
ACCESS

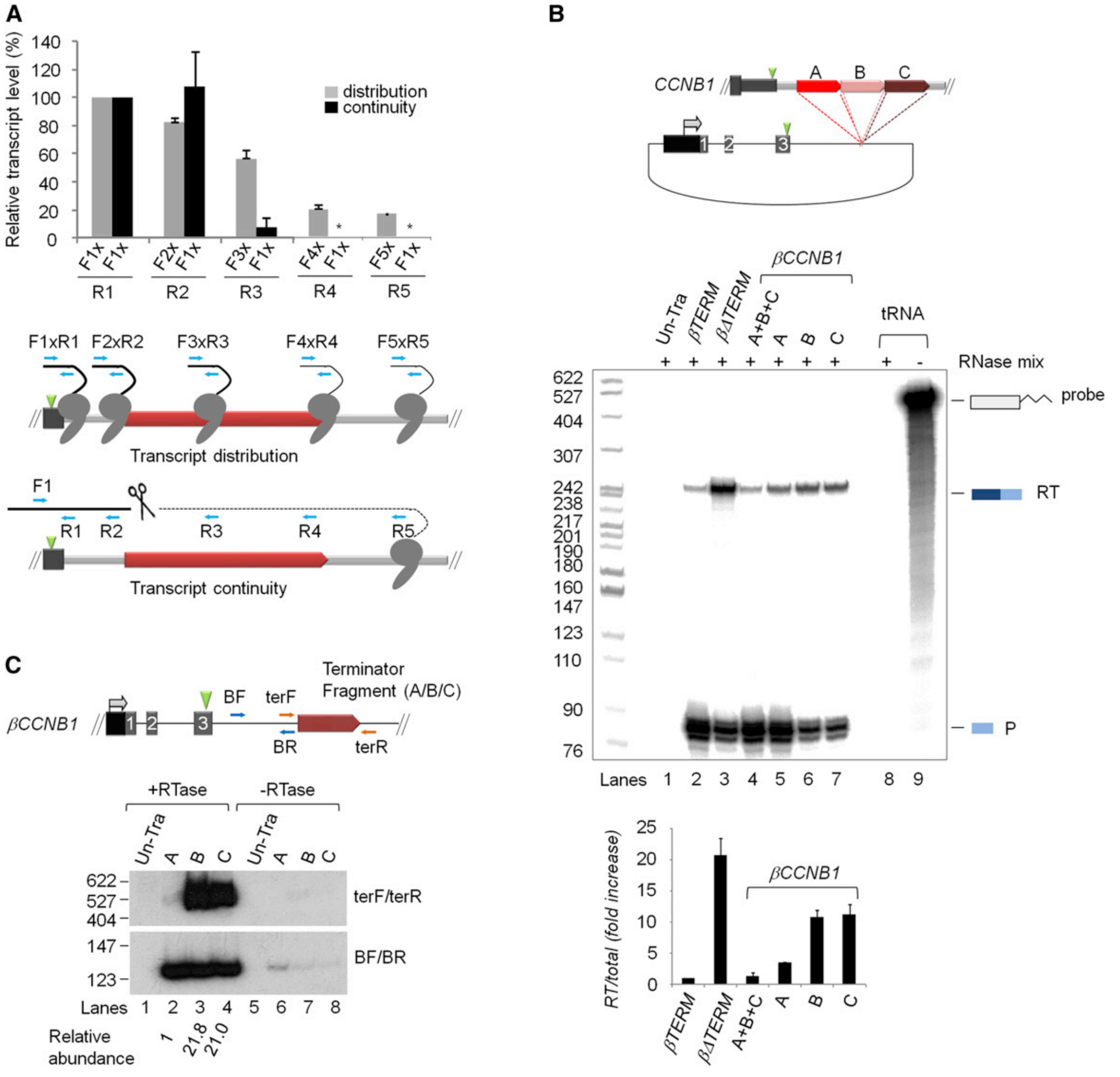

Figure 5. Mapping of CoTC Termination Activity in the *CCNB1* 3′ Flanking Region

(A) Graph showing results of qRT-PCR analysis of transcript distribution (gray bars) and transcript continuity (black bars) at the *CCNB1* gene terminator. Diagrams, below the graph, show primer pairs (blue arrows) used in transcript distribution and transcript continuity analyses. The *CCNB1* poly(A) site (green arrowhead) and terminator element (red bar) are indicated. In the graph, (*) indicates that no PCR product was detected with the indicated primer pairs. Error bars represent the results of three experimental repeats.

(B) RPA of *CCNB1* terminator fragments. In the diagram of the βΔTERM reporter plasmid, dashed red lines indicate the insertion site of *CCNB1* terminator fragments (labeled colored bars). Lane 1, untransfected cells, lanes 2–7 transfected cells. Control RNase digestion of the riboprobe is shown in lane 8 ($tRNA^{+}$) beside undigested riboprobe ($tRNA^{-}$, lane 9). For each sample RT and P protection products were quantified by PhosphoImage analysis and the relative abundance of the RT product (RT/Total) was calculated and displayed in the graph below the data panel. Error bars represent the results of three experimental repeats.

(C) qRT-PCR analysis of the continuity of *CCNB1* terminator subfragment transcripts. In the diagram, the location of PCR primers (red and blue arrows), relative to terminator fragments (red bar) is shown. Lane 1, qRT-PCR of untransfected cells. Lanes 2–4, cells transfected with *CCNB1* terminator fragment constructs. Lanes 5–8, control qRT-PCR of −RTase samples. cDNA in all samples was amplified using 15 PCR cycles (data not shown), which was determined to be within the linear range and therefore accurately reflects RNA abundance.

See also Figure S2.

transcription termination is not clear. Thus our bioinformatic analysis shows that CoTC terminators are not characterized by a simple sequence motif and indicates that factors apart from DNA sequence are involved in the CoTC termination process.

DISCUSSION

Transcription termination is an important, yet relatively overlooked, aspect of the Pol II transcription cycle. Major reasons for this are the considerable technical difficulties involved in

the analysis of nascent Pol II transcripts and the fact that Pol II termination has not as yet, been recapitulated in vitro. However, the finding that Pol II transcription termination on the human β-globin gene, which occurs by the CoTC terminator mechanism (Dye and Proudfoot, 2001), involves release of β-globin pre-mRNA from the chromatin template to the nucleoplasm (West et al., 2008) has enabled us to develop a method for identification of Pol II terminator elements. We have conducted IP of nucleoplasmic RNA, using antibody against the pre-mRNA processing factor CstF-64, to select pre-mRNAs that are released from transcription sites prior to 3′ end processing. Mass sequencing of such CstF-64 interacting pre-mRNAs enabled the identification of 78 candidate genes for the CoTC termination pathway. Detailed transcriptional analysis of the 3′ flanking regions of a randomly selected subset of five candidate genes (*CCNB1*, *PTCH2*, *WDR13*, *THOC2*, and *AKIRIN1*), resulted in the identification of CoTC terminator elements located 0.5–2 kb downstream of the candidate gene poly(A) sites. Each terminator element promotes efficient Pol II termination with the most potent, from the *CCNB1* and *PTCH2* gene 3′ flanking regions, mediating 100% Pol II termination in nuclear run on assays. From these results we predict that the remaining 73 candidate genes contain CoTC terminators within their 3′ flanking regions.

Although we have identified CoTC terminators at a number of gene loci, our data indicate that this number is limited because we have not reached saturation in identification of unprocessed pre-mRNAs in the nucleoplasm. This is possibly due to both the relatively low abundance of these species and gene-specific variation in the strength of the poly(A) site-CstF-64 interaction (Takagaki and Manley, 1997; Martin et al., 2012). Thus it is likely that many more protein coding genes employ the CoTC termination mechanism. Supporting evidence for this suggestion comes from an electron microscopic study of Pol II transcription in *Drosophila*. In this study, of over 100 unidentified Pol II transcribed genes, it was found that Pol II termination and pre-mRNA release occurred prior to pre-mRNA 3′ end processing for 64% of these genes (Osheim et al., 2002). Although the mechanism of Pol II termination at these gene loci is unknown, the observation of abundant released pre-mRNA is suggestive of a CoTC-type termination pathway.

In order to understand more about the possible role of DNA sequence in the CoTC termination mechanism we conducted a detailed bioinformatic analysis of the 3′ flanking regions (0–2 kb downstream of the CLIP-seq sites) of all 78 CoTC candidate genes. From this analysis, we found that these regions are more AT- and T-rich than equally sized regions downstream of the annotated pA sites of other protein coding genes. Although a search for known transcription factor DNA binding motifs in the candidate gene 3′ flanking regions, using the TRANSFAC database (1,665 binding motif matrices), revealed no matches, a search for potential novel sequence motifs using MEME software (Bailey and Elkan, 1994) did identify a number of weak motifs in a subset of candidate gene 3′ flanking regions. Further work will be required to determine the importance of these sequence motifs in the CoTC termination process. Combining these data with our understanding of the role of AT-rich sequences in the human β-globin terminator (Dye and Proudfoot, 2001: White et al., 2013) enables us to state that CoTC terminator sequences are complex and are not characterized by a simple sequence motif. An interesting possibility is that the length and sequence composition of CoTC terminator elements may have affects on nucleosome organization (Kaplan et al., 2009) that may be instrumental in the Pol II termination process.

Apart from mapping CoTC terminators our CLIP-seq strategy illuminates another termination pathway at endogenous gene loci. Analysis of pre-mRNA from the *GAPDH*, *PKM2*, and *ENO1* genes (that were not selected by IP of nucleoplasmic pre-mRNA) shows that for these genes, cotranscriptional poly(A) site cleavage precedes release of Pol II from the chromatin template. Such an order of events corresponds to the pausing model of transcription termination where it is envisioned that G-rich sequences, located immediately downstream of the poly(A) site, cause a transient pause in Pol II progression that effectively enhances 3′ end processing (Gromak et al., 2006). In agreement with this model the *GAPDH*, *PKM2*, and *ENO1* genes all have enrichment of G residues in the post poly(A) site region, which correlates with a recent chromatin immunoprecipitation (ChIP) analysis showing significant Pol II accumulation at the 3′ ends of the *GAPDH* and *ENO1* genes (Brannan et al., 2012).

Although we have discussed Pol II termination in terms of CoTC and pausing models (Figure 6) this may be an oversimplification. Results herein and in previous analyses of CoTC sequences, show significant variation in CoTC terminator efficiency (Dye and Proudfoot, 2001; Plant et al., 2005; West et al., 2006). Considering the sequence-specificity of CoTC (AT-rich) and pause (G-rich) terminator elements, it is likely that the relative contribution of each termination mechanism, at individual gene loci, is directed by 3′ flanking region sequence composition. This leads us to speculate that, especially in the light of the observation that CoTC termination can enhance levels of gene expression (West and Proudfoot, 2009), gene-specific 3′ flanking region sequence composition could have subtle, but important, effects on gene expression.

This study marks a successful attempt to map Pol II terminator elements at endogenous gene loci. It has enabled the characterization of a significant number of CoTC terminator elements, which we predict to be a common feature of mammalian genes, and the visualization of a different termination mechanism, possibly pause-type, occurring at gene loci that were not selected using the CLIP-seq methodology. We anticipate that application of the range of techniques described herein will enable the definition of many more terminator elements and lead to a deeper understanding of mammalian Pol II termination pathways and their roles in gene expression.

EXPERIMENTAL PROCEDURES

PCR Primers and RNA Linkers

A list of oligonucleotide sequences used as PCR primers and RNA linkers is given in Table S1.

Plasmid Constructions

pTat, βTERM, βΔTERM, and β4-7 (formerly βΔ5–7, βΔ5–10, and βΔ8–10) have been described previously (Dye and Proudfoot, 2001). βCCNB1, βPTCH2,

Cell PRESS Open ACCESS

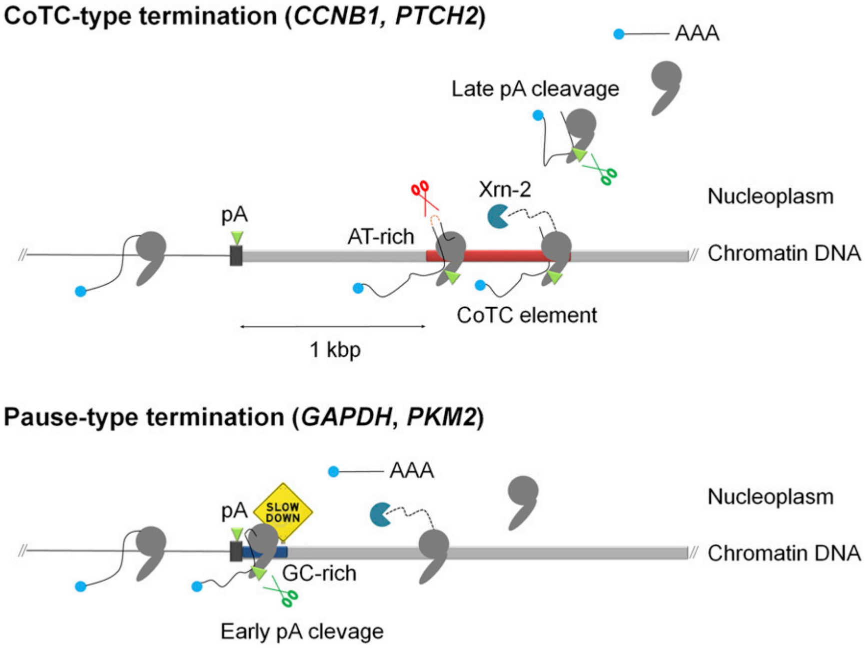

Figure 6. Diagram of CoTC and Pause-Type Pol II Termination Pathways

In the CoTC termination pathway transcripts of AT-rich terminator elements (red bar) are cleaved by CoTC activity (red scissors) promoting Pol II release before poly(A) site cleavage, mediated by the 3′ processing complex (green scissors). In the pause-type termination pathway, Pol II transcriptional pausing, at G-rich sequences (blue bar), enhances pre-mRNA cleavage at the poly(A) site, leading to Pol II release. Icons and symbols as in Figure 1A.

βAKIRIN1, βTHOC2, βWDR13, and βCCNB1/A/B and C expression plasmids were made by insertion of genomic PCR fragments, isolated using gene-specific primer sets, into a cloning vector prepared by long range PCR amplification (Barnes, 1994) of βΔTERM with primers BETA43/BETA10.3 using PrimeSTAR HS DNA polymerase (Takara).

Transfection Procedure

Transient transfection of HeLa cells was performed as previously described (West et al., 2008).

Nuclear RNA Fractionation

Nuclear RNA fractionation was performed as previously described (West et al., 2008).

RNA Immunoprecipitation

HeLa cells on 10 cm plates were irradiated with 254 nm UV (300 mJ/cm^2) prior to nuclear fractionation. RNA in the nucleoplasm fraction was immunoprecipitated with either rabbit IgG or CstF-64 antibody (Cambridge Bioscience) conjugated to Protein G Dynabeads (Invitrogen). Following 1 hr incubation at 4°C, beads were washed four times with NET-2 buffer (10 mM Tris-HCl pH7.5, 150 mM NaCl, 0.05% NP-40) and precipitated RNA eluted in Trizol (Invitrogen).

HITS Library Preparation and Data Processing

HITS library preparation was performed as described (Licatalosi et al., 2008). Samples from three independent repeats of CstF-64 CLIP-seq were submitted to high-throughput sequencing from the 5′ ends using the Illumina Hi-Seq 100 nt single-end reads protocol (Source Bioscience). Repeats 1 and 2 were multiplexed and sequenced together in the same lane. During pre-processing, samples 1 and 2 were demultiplexed using barcodes; low quality reads (mean Q < 30 within the first 50 bases) were removed and 3′ sequences matching ligated adaptors or putative oligo-A tails trimmed. Reads shorter than 24 nt were discarded and longer reads trimmed to 50 nt before Bowtie alignment to the hg18 assembly of the human genome allowing for three mismatches. From a total of 97.5 M reads (40.1 M, 32.9 M and 24.5 M, in repeats 1, 2, and 3, respectively), 49.8 M aligned, of which 38.5 M (13.1 M, 10.9 M, and 13.8 M in repeats 1, 2, and 3, respectively) matched unique sites (only these were considered in the subsequent analysis). Each of the experimental repeats resulted in a high level of duplication indicated by many reads aligned to the same genomic location, probably due to the low amount of pre-mRNA targets recovered from the nucleoplasm in the CLIP experiment. To avoid bias PCR duplicates were removed and each unique read-alignment location was considered only once, resulting in 9,658 (3,410, 2,765, and 3,483, in repeats 1, 2, and 3, respectively) read-alignment sites. To maximize sensitivity reads from the three repeats were pooled for subsequent analysis. Overlapping read-alignments (extended by 100 bp) were merged to "regions." A total of 1,285 regions, supported by at least two independent read-alignments, were considered significant. Many regions were identified in only one repeat, indicating that the experiment was far from saturation in detection of nucleoplasmic CstF-64 binding targets. To determine their genomic distribution, significant regions were related to NCBI RNA reference sequences (RefSeq) and short RNA gene (RNA genes) annotation tracks, downloaded from the UCSC genome browser. To identify genes that employ the CoTC termination pathway we selected pre-mRNAs with a CstF-64 CLIP region in 3′ UTRs, extended by 200 bp downstream (to account for variability in poly(A) site usage and imprecision in transcript-end annotation).

RT-PCR and qRT-PCR

cDNA was synthesized using Superscript III (Invitrogen). DNA amplification was performed using Go-Taq DNA polymerase (Promega). When conducting qRT-PCR, PCR products were amplified with [α-^{32}P]dCTP (Perkin Elmer). PCR products were applied to 6% polyacrylamide gels and radioactive signals quantified by PhosphoImager (Fuji).

RNase Protection Analysis

RNase protection analysis is as described previously (Plant et al., 2005).

NRO Analysis and Single-Stranded DNA probes

NRO analysis and single-stranded M13 probes used are as described previously (West et al., 2008). Quantitation of NRO hybridization signals by PhosphoImager analysis is based on the average of multiple experiments after subtraction of background signal, shown by probe M.

ACCESSION NUMBERS

The sequences generated for this work have been deposited in the Array Express Archive under accession number E-MTAB-1375 (http://www.ebi.ac.uk/arrayexpress/experiments/E-MTAB-1375).

SUPPLEMENTAL INFORMATION

Supplemental Information includes four figures and one table and can be found with this article online at http://dx.doi.org/10.1016/j.celrep.2013.03.012.

LICENSING INFORMATION

This is an open-access article distributed under the terms of the Creative Commons Attribution License, which permits unrestricted use, distribution, and reproduction in any medium, provided the original author and source are credited.

ACKNOWLEDGMENTS

This work was supported by a Programme Grant from the Wellcome Trust to N.J.P., grants from the Wellcome Trust and EPA Trust to S.M., and a long term fellowship from the Uehara Memorial Foundation to T.N.

Received: November 1, 2012
Revised: February 12, 2013
Accepted: March 12, 2013
Published: April 4, 2013

REFERENCES

Bailey, T.L., and Elkan, C. (1994). Fitting a mixture model by expectation maximization to discover motifs in biopolymers. Proceedings of the 2nd International Conference on Intelligent Systems for Molecular Biology (Menlo Park, CA: AAAI Press), pp. 28–36.

Barnes, W.M. (1994). PCR amplification of up to 35-kb DNA with high fidelity and high yield from lambda bacteriophage templates. Proc. Natl. Acad. Sci. USA *91*, 2216–2220.

Brannan, K., Kim, H., Erickson, B., Glover-Cutter, K., Kim, S., Fong, N., Kiemele, L., Hansen, K., Davis, R., Lykke-Andersen, J., and Bentley, D.L. (2012). mRNA decapping factors and the exonuclease Xrn2 function in widespread premature termination of RNA polymerase II transcription. Mol. Cell *46*, 311–324.

Connelly, S., and Manley, J.L. (1988). A functional mRNA polyadenylation signal is required for transcription termination by RNA polymerase II. Genes Dev. *2*, 440–452.

Dye, M.J., and Proudfoot, N.J. (2001). Multiple transcript cleavage precedes polymerase release in termination by RNA polymerase II. Cell *105*, 669–681.

Gromak, N., West, S., and Proudfoot, N.J. (2006). Pause sites promote transcriptional termination of mammalian RNA polymerase II. Mol. Cell. Biol. *26*, 3986–3996.

Gupta, R., Bhattacharyya, A., Agosto-Perez, F.J., Wickramasinghe, P., and Davuluri, R.V. (2011). MPromDb update 2010: an integrated resource for annotation and visualization of mammalian gene promoters and ChIP-seq experimental data. Nucleic Acids Res. *39*(Database issue), D92–D97.

Kaplan, N., Moore, I.K., Fondufe-Mittendorf, Y., Gossett, A.J., Tillo, D., Field, Y., LeProust, E.M., Hughes, T.R., Lieb, J.D., Widom, J., and Segal, E. (2009). The DNA-encoded nucleosome organization of a eukaryotic genome. Nature *458*, 362–366.

Kapranov, P., Cheng, J., Dike, S., Nix, D.A., Duttagupta, R., Willingham, A.T., Stadler, P.F., Hertel, J., Hackermüller, J., Hofacker, I.L., et al. (2007). RNA maps reveal new RNA classes and a possible function for pervasive transcription. Science *316*, 1484–1488.

Kuehner, J.N., Pearson, E.L., and Moore, C. (2011). Unravelling the means to an end: RNA polymerase II transcription termination. Nat. Rev. Mol. Cell Biol. *12*, 283–294.

Licatalosi, D.D., Mele, A., Fak, J.J., Ule, J., Kayikci, M., Chi, S.W., Clark, T.A., Schweitzer, A.C., Blume, J.E., Wang, X., et al. (2008). HITS-CLIP yields genome-wide insights into brain alternative RNA processing. Nature *456*, 464–469.

MacDonald, C.C., Wilusz, J., and Shenk, T. (1994). The 64-kilodalton subunit of the CstF polyadenylation factor binds to pre-mRNAs downstream of the cleavage site and influences cleavage site location. Mol. Cell. Biol. *14*, 6647–6654.

Martin, G., Gruber, A.R., Keller, W., and Zavolan, M. (2012). Genome-wide analysis of pre-mRNA 3′ end processing reveals a decisive role of human cleavage factor I in the regulation of 3′ UTR length. Cell Rep *1*, 753–763.

Osheim, Y.N., Sikes, M.L., and Beyer, A.L. (2002). EM visualization of Pol II genes in Drosophila: most genes terminate without prior 3′ end cleavage of nascent transcripts. Chromosoma *111*, 1–12.

Plant, K.E., Dye, M.J., Lafaille, C., and Proudfoot, N.J. (2005). Strong polyadenylation and weak pausing combine to cause efficient termination of transcription in the human Ggamma-globin gene. Mol. Cell. Biol. *25*, 3276–3285.

Proudfoot, N.J. (1989). How RNA polymerase II terminates transcription in higher eukaryotes. Trends Biochem. Sci. *14*, 105–110.

Proudfoot, N.J. (2011). Ending the message: poly(A) signals then and now. Genes Dev. *25*, 1770–1782.

Proudfoot, N.J., Furger, A., and Dye, M.J. (2002). Integrating mRNA processing with transcription. Cell *108*, 501–512.

Richard, P., and Manley, J.L. (2009). Transcription termination by nuclear RNA polymerases. Genes Dev. *23*, 1247–1269.

Shi, Y., Di Giammartino, D.C., Taylor, D., Sarkeshik, A., Rice, W.J., Yates, J.R., 3rd, Frank, J., and Manley, J.L. (2009). Molecular architecture of the human pre-mRNA 3′ processing complex. Mol. Cell *33*, 365–376.

Takagaki, Y., and Manley, J.L. (1997). RNA recognition by the human polyadenylation factor CstF. Mol. Cell. Biol. *17*, 3907–3914.

Tantravahi, J., Alvira, M., and Falck-Pedersen, E. (1993). Characterization of the mouse beta maj globin transcription termination region: a spacing sequence is required between the poly(A) signal sequence and multiple downstream termination elements. Mol. Cell. Biol. *13*, 578–587.

West, S., and Proudfoot, N.J. (2009). Transcriptional termination enhances protein expression in human cells. Mol. Cell *33*, 354–364.

West, S., Gromak, N., and Proudfoot, N.J. (2004). Human 5′ —> 3′ exonuclease Xrn2 promotes transcription termination at co-transcriptional cleavage sites. Nature *432*, 522–525.

West, S., Zaret, K., and Proudfoot, N.J. (2006). Transcriptional termination sequences in the mouse serum albumin gene. RNA *12*, 655–665.

West, S., Proudfoot, N.J., and Dye, M.J. (2008). Molecular dissection of mammalian RNA polymerase II transcriptional termination. Mol. Cell *29*, 600–610.

White, E., Kamieniarz-Gdula, K., Dye, M.J., and Proudfoot, N.J. (2013). AT-rich sequence elements promote nascent transcript cleavage leading to RNA polymerase II termination. Nucleic Acids Res. *41*, 1797–1806.

Whitelaw, E., and Proudfoot, N. (1986). Alpha-thalassaemia caused by a poly(A) site mutation reveals that transcriptional termination is linked to 3′ end processing in the human alpha 2 globin gene. EMBO J. *5*, 2915–2922.

Supplemental Information

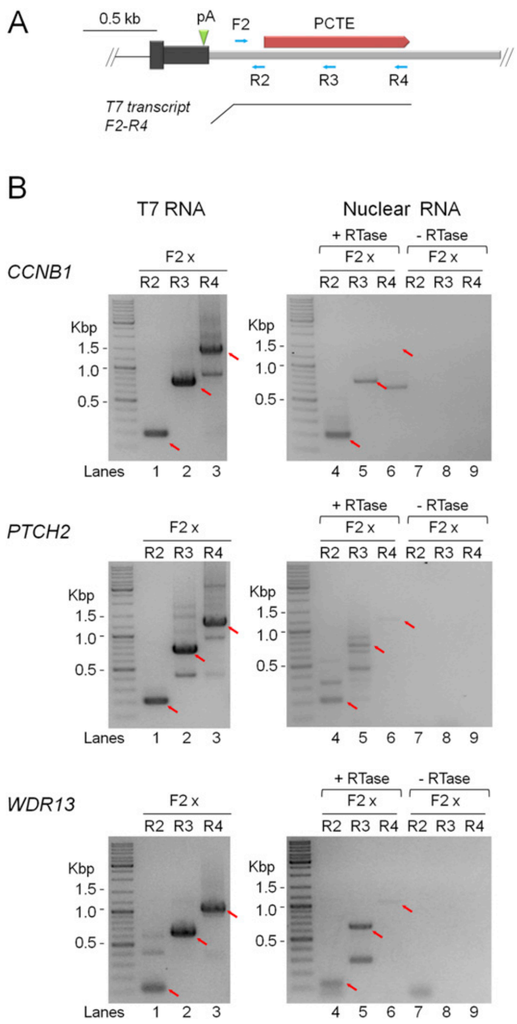

Figure S1. Control Experiment to Test RT-PCR Efficiency on PCTE Transcripts, Related to Figure 3

(A) Diagram showing candidate gene PCTEs (red box), control T7 transcripts (black line) and PCR primers (blue arrows). The gene specific PCR primer sets used are as in Figure 3.

(B) Data from RT-PCR analysis of *CCNB1*, *PTCH2* and *WDR13* PCTEs. Full length T7 PCTE transcripts and PCTE transcripts from HeLa cell nuclei were reverse transcribed with random primers. The resulting cDNAs were PCR amplified (26 PCR cycles for T7 RNA templated cDNA and 32 cycles for HeLa nuclear RNA templated cDNA) using indicated gene specific primer pairs. Lanes 1-3, RT-PCR products from T7 PCTE transcripts. The presence of the prominent, expected, PCR products (indicated by red arrows) in each lane indicates that the control T7 PCTE transcripts are efficiently reverse transcribed and PCR amplified, irrespective of their length. Lanes 4-6, RT-PCR products from HeLa nuclear PCTE transcripts. Prominent bands, corresponding to the 5′ end of PCTE transcripts, are detected in lane 4 for all samples, however RT-PCR products representing longer PCTE transcripts, in lanes 5 and 6, are of lower abundance and in some cases entirely absent (e.g., *CCNB1*, lane 6). Comparison of HeLa nuclear PCTE transcript RT-PCR products (in lanes 4-6) with RT-PCR products from control T7 transcripts (lanes 1-3) shows that detection of longer endogenous nuclear transcripts is significantly lower than for the corresponding T7 control transcripts. This result indicates that the longer PCTE transcripts are less abundant in HeLa nuclei, due to transcript termination or transcript cleavage, rather than template length dependent decrease in RT-PCR efficiency. The absence of PCR products in control lanes 7-9 (-RTase) confirms the absence of contaminating DNA in these experiments.

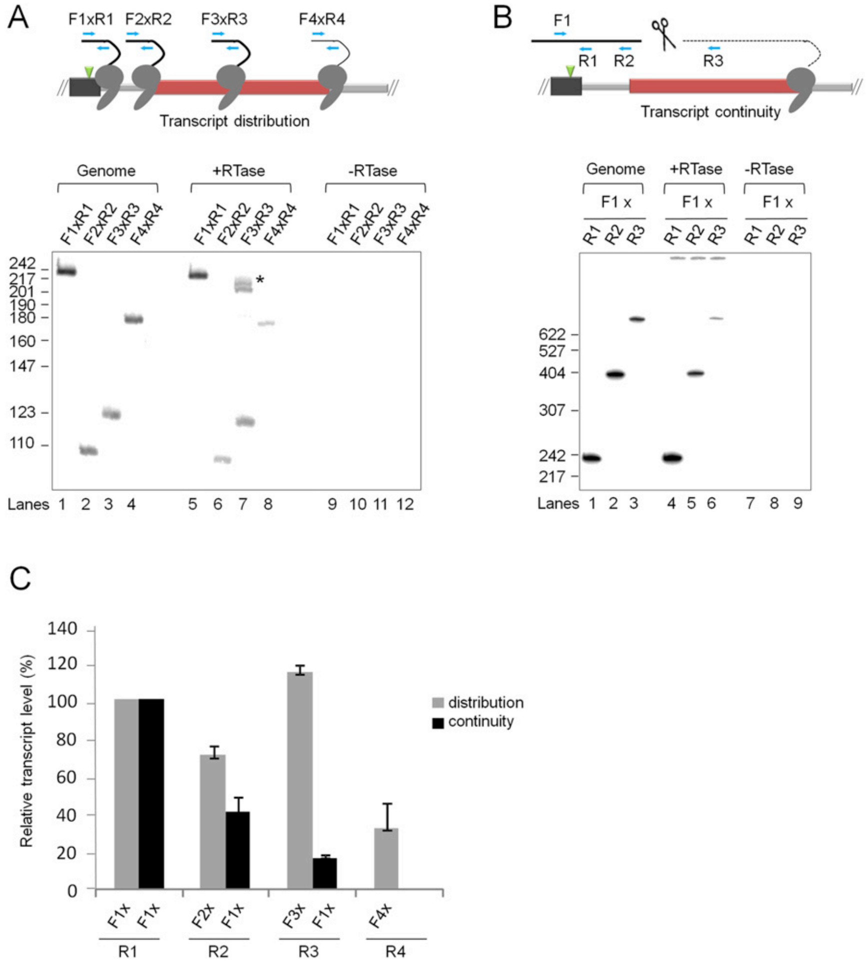

Figure S2. *WDR13* 3′ Flanking Region Transcript Distribution and Continuity, Related to Figure 5

(A) Transcript distribution. The diagram above the data panel shows the *WDR13* 3′ flanking region, including the poly(A) site (green arrowhead) and terminator element (red box). Primers used in qRT-PCR analysis are indicated by blue arrows. Lanes 1-4, control qPCR amplification of genomic DNA. Lanes 5-9, qRT-PCR of *WDR13* 3′ flanking region transcripts. Lanes 9-12, control qRT-PCR of -RTase samples. (*) the high Mw qRT-PCR products in lane 7 are non specific RT-PCR products of unknown origin.

(B) Transcript continuity. Diagram labeled as in (A), with addition of scissors indicating CoTC. Lanes 1-3, control qPCR amplification of genomic DNA. Lanes 4-6, qRT-PCR of *WDR13* 3′ flanking region transcripts. Lanes 7-9, control qRT-PCR of -RTase samples.

(C) Bar graph showing quantitative analysis of *WDR13* 3′ flanking region transcript distribution (gray bars) and continuity (black bars). Transcript abundance was calculated by phosphoImage quantitation of radioactive qRT-PCR products (+RTase lanes in (A) and (B)), normalized to products resulting from qPCR amplification of corresponding genomic DNA sequences (Genome lanes in (A) and (B)). The transcript distribution data show that transcript abundance decreases to low levels at the 3′ end of the terminator element, presumably due to the occurrence of Pol II termination. The transcript continuity data shows that although some continuous transcripts are detected at the 5′ end of the terminator, the level of continuous transcripts decreases markedly beyond this point. These data indicate that nascent transcripts of the 5′ end of the *WDR13* terminator are co-transcriptionally cleaved and, when combined with results from the measurement of transcript distribution above, indicate that transcript cleavage precedes Pol II termination occurring throughout the *WDR13* terminator. This profile of transcript discontinuity followed by Pol II termination is similar to that described for the human β-globin gene terminator (Dye and Proudfoot, 2001) and *CCNB1* terminator (Figure 5A) and is indicative of the presence of the CoTC termination mechanism at the *WDR13* gene locus. Error bars represent the results of three experimental repeats.

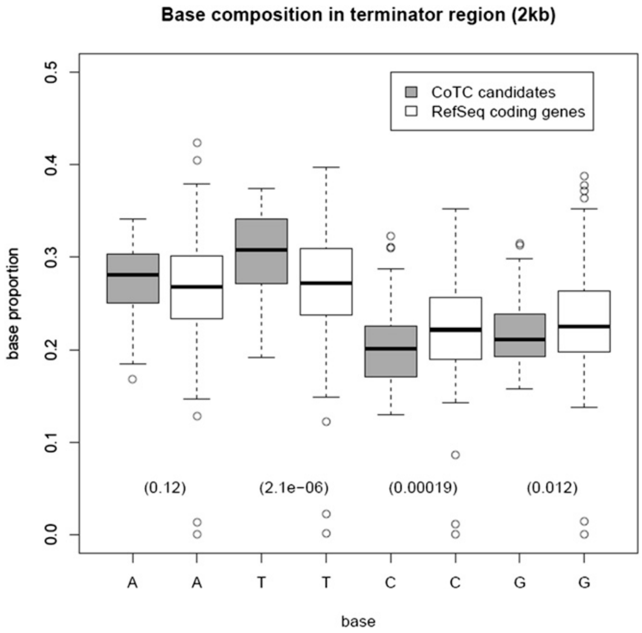

Figure S3. Nucleotide Composition of CoTC Terminators, Related to Results

Comparison of the nucleotide composition of the sense strand of the putative CoTC terminator regions; 2Kb downstream of the CLIP-seq sites, for 78 CoTC candidate genes (dark gray) and 2Kb downstream of annotated pA sites for 500 randomly selected protein coding genes (white). Boxes show upper and lower quartiles, black lines represent medians. t test p-values are shown in parenthesis below.

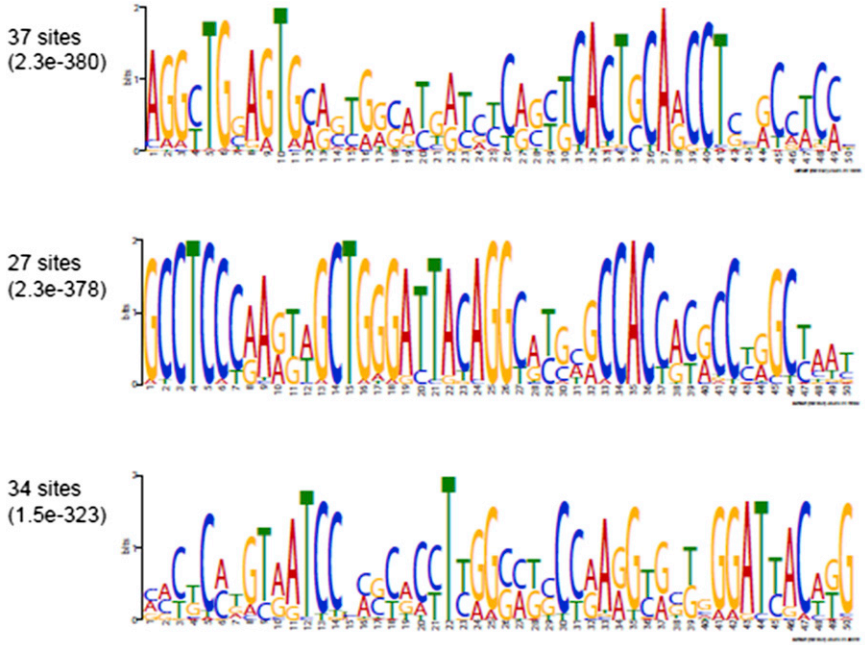

Figure S4. Result of Search for Potential Novel Sequence Motifs Using MEME Software, Related to Results and Discussion
Putative CoTC terminator regions (2Kb downstream of the CLIP-seq sites for the 78 CoTC candidate genes) were analyzed for the presence of shared motifs using position specific priors based on a negative sequence set (2Kb downstream of annotated pA sites for 500 randomly selected protein coding genes). The top three motifs are shown, together with p-values and number of sequences in which they were found. While as expected several motifs are identified by this approach, their relevance is questionable as they only occur in a subset of candidates ('zoops' mode). No strong motif was present in all candidates ('oops' mode).
